# Different responses of foliar nutrient resorption efficiency in two dominant species to grazing in the desert steppe

**DOI:** 10.1038/s41598-024-53574-3

**Published:** 2024-02-19

**Authors:** Qingge Zhao, Yuhan Zhang, Yunbo Wang, Guodong Han

**Affiliations:** 1 College of Grassland, Resources and Environment, Key Laboratory of Grassland Resources of the Ministry of Education, Inner Mongolia Agricultural University, Hohhot, 010011 China; 2 Key Laboratory of Grassland Resources, Ministry of Education, College of Grassland, Resources and Environment, Inner Mongolia Agricultural University, Hohhot, 010011 China; 3 Forestry and Grassland Work Station of Inner Mongolia, Hohhot, 010011 China

**Keywords:** Ecosystem ecology, Grassland ecology

## Abstract

Nitrogen and phosphorus resorption (NRE and PRE) is a critical nutrient conservation mechanism maintaining plant growth in already disturbed barren ecosystems. The complexity of plant nutrient resorption variations in long-term grazing regions is regulated by plant traits, nutritional utilization strategies, and soil conditions following changes in grazing patterns. Therefore, a detailed investigation into their underlying mechanism is still required. Here we investigated leaf nutrient concentration and resorption in dominant species *Cleistogenes songorica* (*C. squarrosa*) and *Stipa breviflora* (*S. breviflora*) response to 15-years continuous grazing (moderate and heavy grazing) in desert steppe. Moderate grazing enhanced green leaf N and P content in *C. songorica* and partially increased N content in *S. breviflora*. Heavy grazing consistently increased N content in *C. songorica*, but its P content as well as N and P content in *S. breviflora* were largely stable. Moderate grazing enhanced NRE but unaffected PRE in both *S. breviflora* and *C. songorica*. Heavy grazing reduced NRE and PRE in *C. songorica*. Although soil variables (nutrients and moisture) did not affect foliar nutrients, it’s a key driver of nutrient resorption efficiency. Of all measured influence factors, soil moisture is the one most important and negatively correlated with NRE and PRE in *S. breviflora*. While it was not observed in *C. songorica*. In *S. breviflora*, its NRE was adversely linked with soil N, in addition, both NRE and PRE were positively associated with green leaf nutrients. Senesced leaf nutrients are the predominant factor influencing nutrient resorption efficiency in *C. songorica*, which were adversely associated. Overall, our results indicate significant variations in nutrient resorption efficiency patterns between the two dominant species due to divergent plant adaptation strategies to grazing and the local environment. The foliar nutritional status and soil conditions may play significant roles in regulating nutrient resorption in arid long-term grazing desert steppe.

## Introduction

As the essential nutrients, Nitrogen (N) and phosphorus (P) play a crucial role in supporting plant functionality and growth^1^. Large demand and limited source make them the primary limiting factors in terrestrial ecosystems^2^. Generally, the nutrient requirements of plants are partly (or completely) met through the supply of extrinsic resources (e.g., soil, parent material, and atmosphere) and intrinsic conservation mechanisms (e.g., nutrient resorption). Nutrient resorption, the movement of nutrients from senescing tissues back to surviving tissues, would contribute to increasing nutrient use efficiency and decreasing dependence on external nutrient supply^3–5^. In most instances, the impact of increased soil nutrient bioavailability on plant growth appeared to be more pronounced than that of nutrient resorption. Nevertheless, the significance of nutrient resorption in plants has been comparatively underestimated in certain arid grassland for the difficult challenges associated with nutrient release and absorption. Nutrient reuse and storage from senescing leaves can maximize plant adaptability, more particularly in nutrient poor conditions are broadly validated^6,7^. Unfortunately, questions still remain to be addressed as nutrient resorption efficiency response to anthropogenic disturbances such as grazing, particularly in the barren desert steppe.

Herbivores are suggested to directly or indirectly regulate plants’ nutrient allocation and resorption efficiency in numerous studies^8–10^. However, the combined effects of selective feeding, trampling, and excretion on nutrient resorption efficiency make it hard to predict its response to grazing. Such as the one by Zhang et al.^9^ proposed that both N resorption efficiency (NRE) and P resorption efficiency (PRE) are enhanced as a result of grazing-induced plants’ regrowth and/or accelerated nutrient cycling in grassland ecosystems. Moreover, inhibiting or even no effect on nutrient resorption by grazing has been detected^11–13^. These inconsistent results are due largely to the interactions of plant traits (e.g., chemical and morphological traits, and species-specific changes) and grazing patterns (e.g., grazers, grazing intensity). Firstly, the grazed livestock can modify plant-derived nutrient inputs through selective foraging-induced changes in litter composition^14,15^. Additionally, trampling and excreta deposition resulting from grazing activities also influence soil nutrient cycling processes and supply capacity in grassland ecosystems^16,17^, ultimately leading to variations in nutrient resorption efficiency. Secondly, the adaptive strategy of grassland vegetation may transform in response to grazing-induced changes in abiotic and biotic environments^18^, undoubtedly affecting the internal nutrient allocation and/or resorption efficiency^11^. The response of nutrient resorption to these expected feedbacks appear to be complicated and unpredictable in desert steppe, the underlying mechanisms need further investigation^9^.

Nutrient resorption efficiency variations may arise from a variety of factors, including climate and soil conditions, as well as plant physiological characteristics that can act as a strong force on it^7^. In which soil conditions such as hydrological and chemical variables, and leaf traits lead to nutrient resorption variations conditionally^6,19^. The connection between nutrient resorption efficiency and soil nutrients is still an ongoing debate. They were observed negatively correlated based on the global dataset^20^ as well as studies in nutrient poor sites^21^, which is widely supported. On the other hand, no response of nutrient resorption to soil nutrients was detected^1,22^. The divergent associations should be overall considered by the environmental nutrient supply and plant adaptability^6,23^. Moreover, water may be a more determinant factor in soil nutrient availability and plant uptake in dry regions^24,25^. A decrease in NRE and PRE was observed with increasing soil moisture in a semi-arid steppe^21^. Furthermore, the result is expected to be differ between species with the adaptation and tolerance to the water-limited conditions^26^. Building on these perspectives, leaf traits (e.g., chemical traits, species-specific changes) should be supposed to be a driver of nutrient resorption apart from the local environment and plant strategies. For instance, foliar chemical traits (e.g., N and P) were observed adversely linked with nutrient resorption efficiency based on a global dataset^27^. Yuan et al.^28^ uncovered conflicting results in the semi-arid areas which may attributed to the activity of chemical compounds in leaves. Therefore, the biotic and abiotic factors influencing nutrient resorption efficiency are necessary to be further investigated to estimate the plants’ nutrient reuse and cycling in desert steppe.

This study investigated the influence of 15-years continuous grazing (moderate and heavy grazing) on the foliar nutrients and resorption efficiency in two dominant species i.e., *Stipa breviflora* (*S. breviflora*; C3 perennial grass) and *Cleistogenes songorica* (*C. songorica*; C4 perennial grass) in desert steppe. Here, we hypothesized that: (1) grazing (MG and HG) would increase NRE, PRE, and green leaf nutrient concentration with potentially interspecific variability; (2) soil moisture and nutrients and foliar chemical traits regulate and negatively correlate with both NRE and PRE in desert steppe.

## Results

### Grazing effects on leaf nutrient and soil properties

**Figure 1:**
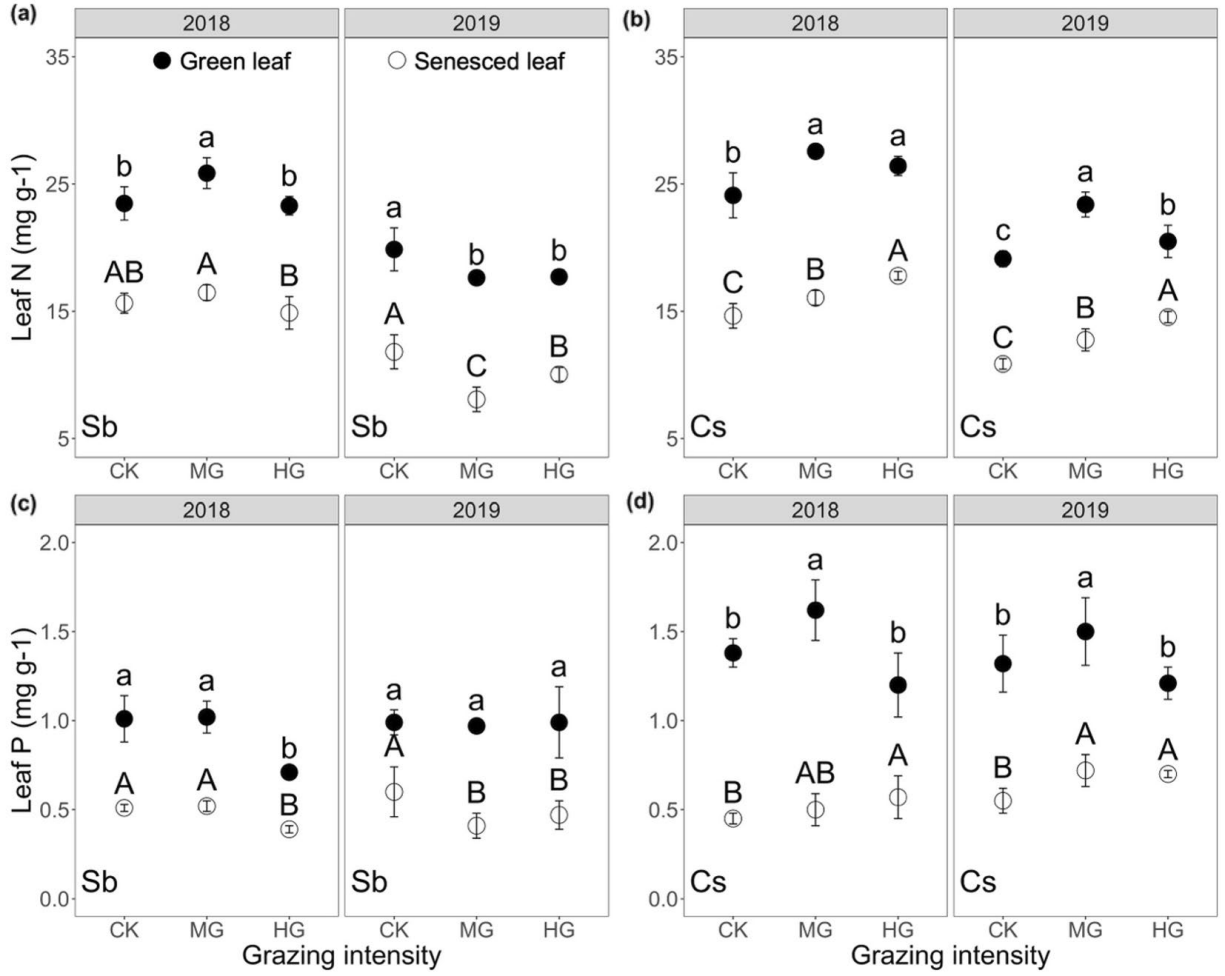
Concentrations of leaf N (mg g^-1^) in (**a**) *S. breviflora* and (**b**) *C. songorica*, and leaf P (mg g^-1^) in (**c**) *S. breviflora* and (**d**) *C. songorica* for 2018 and 2019. Error bars show standard errors (n = 6), with significant differences indicated by different lowercase for green leaf and uppercase letters for senesced leaf among grazing intensities (*p* < 0.05). CK: control check; MG: moderate grazing; HG: heavy grazing.

In both species, green leaf nutrients showed a clear response to grazing treatments (Fig. 1). Compared to control check (CK), moderate grazing (MG) significantly enhanced green leaf N and P content by up to 22% and 17% in *C. songorica*, respectively (Fig. 1b, d; *p* < 0.05), as well as its senesced leaf nutrient showed consistent results with a significant enhancement. Leaf N content in *S. breviflora* response to MG was dependent on interannual differences, with a significant reduction of 11% in green leaf and 30% in senesced leaf in 2019, but a significant 10% increase in apparent green leaf in 2018 (Fig. 1a). In addition, its green leaf P content was unaffected by MG (Fig. 1c).

Heavy grazing (HG) significantly increased N content by around 8% of green leaf and 7% of senesced leaf in *C. songorica*, but green leaf P content was barely affected (Fig. 1b, d). In terms of interannual differences in *S. breviflora*, HG significantly reduced N content by 11% of green leaf and 13% of senesced leaf in 2019, but they were unaffected in 2018 (Fig. 1a). Moreover, its senesced leaf P content was observed to be significantly decreased by up to a quarter in HG, while unaffected in green leaf (Fig. 1c). Both MG and HG barely affected soil total N content (STN), while were observed to influence soil moisture (SM) and soil total phosphorus (STP) (Table 1). Compared to CK, MG significantly decreased SM in 2019 and STP in 2018 by 10% and 13%, respectively. The SM exhibited a clear response to HG, with a significant decrease of 13% in 2019.

**Table 1:** Soil moisture and nutrients in relation to grazing intensity (CK, MG, HG).

Year	Grazing	SM (%)	STN (g kg^-1^)	STP (g kg^-1^)
2018	CK	11.9 ± 1.4	1.6 ± 0.1	0.48 ± 0.05**a**
	MG	11.1 ± 0.6	1.4 ± 0.1	0.42 ± 0.02**b**
	HG	11.6 ± 1.3	1.5 ± 0.1	0.47 ± 0.04**a**
2019	CK	6.1 ± 0.5**a**	1.7 ± 0.1	0.44 ± 0.02
	MG	5.5 ± 0.8**b**	1.6 ± 0.1	0.45 ± 0.03
	HG	5.3 ± 0.2**b**	1.6 ± 0.1	0.45 ± 0.02

CK: control (no grazing); MG: moderate grazing (1.82 sheep ha^-1^ corresponding to 4 sheep per plot); HG: heavy grazing (2.71 sheep ha^-1^ corresponding to 6 sheep per plot).

SM: soil moisture; STN: soil total nitrogen; STP: soil total phosphorus.

Data correspond to mean values ± standard errors (n = 6). Different bond lowercase letters represent significant differences among grazing intensities (*p* < 0.05).

### Grazing effects on nutrient resorption efficiency

**Figure 2:**
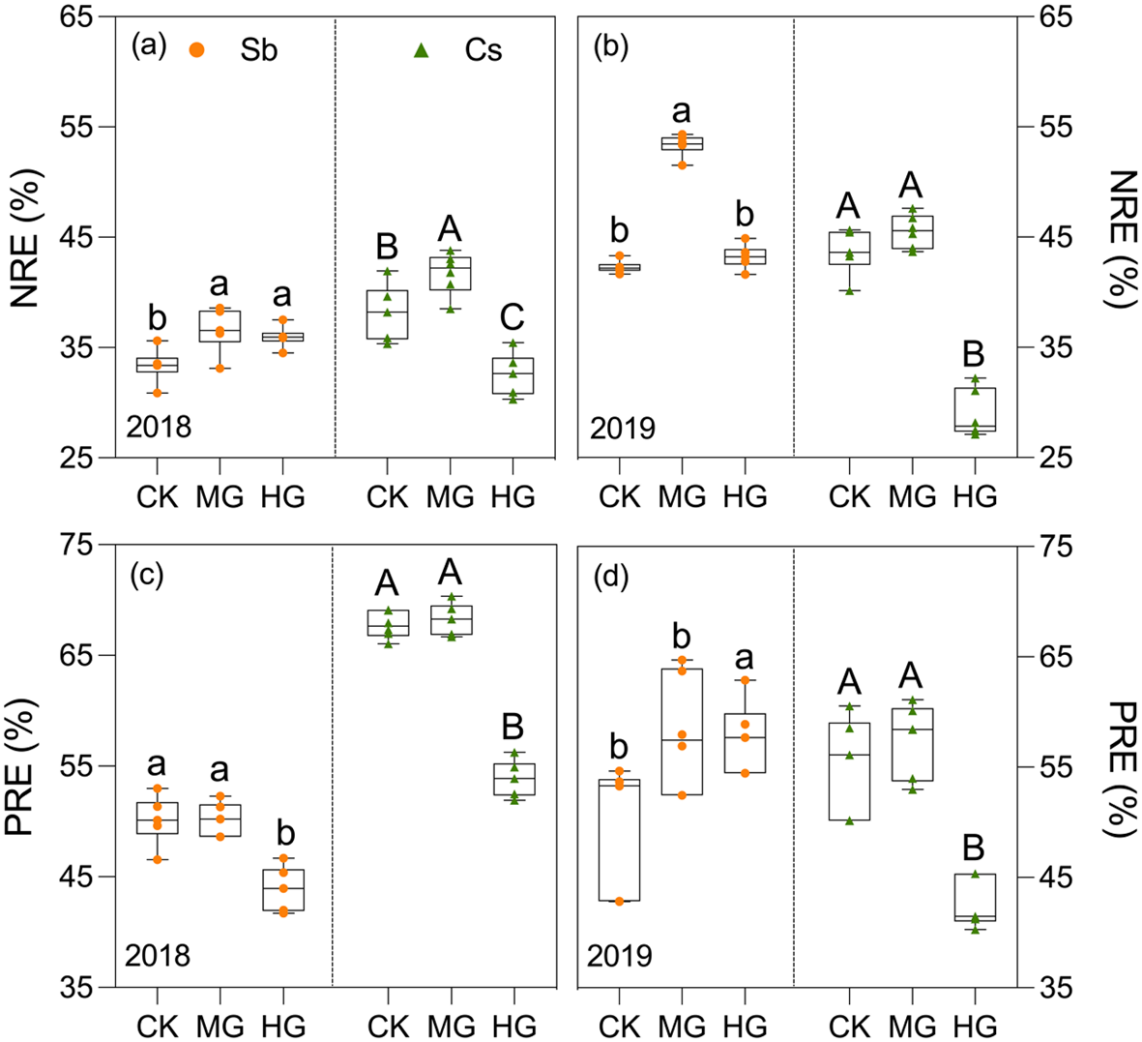
Grazing intensity influenced the NRE (**a**, **b**) and PRE (**c**, **d**) in two dominant species. Error bars correspond to standard errors (n = 6), with different lowercase and uppercase letters indicating significant differences among grazing intensities in *Stipa breviflora* and *Cleistogenes songorica*, respectively (*p* < 0.05). Sb: *Stipa breviflora*; Cs: *Cleistogenes songorica*.

Compared to CK, the MG treatment barely affected PRE while increasing NRE in both species (Fig. 2). Of which NRE was significantly increased by up to 26% in *S. breviflora’*s and 9% in *C. songorica* (Fig. 2a, b; *p* < 0.05). The HG treatment significantly decreased NRE by up to 34% and PRE by up to 20% in *C. songorica*. The PRE in *C. songorica* showed a similar 12% decrease for HG in 2018, while increased by 15% in 2019. Heavy grazing increased NRE in *C. songorica*, significantly by 8% in 2018 (Fig. 2a).

### Relationships among soil properties, leaf nutrient and nutrient resorption efficiency

A linear regression analysis was performed to identify potential relationships between the nutrient resorption efficiency and soil properties and plant nutrient content in each species (Table 2). The NRE in *S. breviflora* was observed to be significant negatively correlated with soil moisture, soil total N and senesced leaf N, but positively correlated with green leaf N (*p* < 0.05). In addition, its PRE was also significant negatively correlated with soil moisture (*p* < 0.05), while positively correlated with green leaf P (*p* < 0.01). *C. songorica*’s NRE and PRE were detected to be significant negatively correlated with senesced leaf N and senesced leaf P, respectively, but its PRE significant positively correlated with green leaf P (*p* < 0.05). Green leaf nutrient (including N and P) content was uncorrelated with soil properties in both species.

**Table 2:** Correlation for green leaf nutrient content and resorption efficiency to soil and plant variables in two species.

Species	Year		Soil			Plant			
			SM	STN	STP	TN_g_	TP_g_	TN_s_	TP_s_
Sb	2018	NRE	− 1.45*	− 15.67*	–	2.04*	–	-0.20**	–
		PRE	− 1.48**	–	− 35.29*	–	10.48**	–	− 20.00
		TN_g_	− 0.18	− 1.65	-	–	–	–	–
		TP_g_	− 2.00	–	-0.11	–	–	–	–
	2019	NRE	− 5.00**	− 33.26*	–	2.51*	–	− 0.18*	–
		PRE	− 6.20*	–	− 233.42	–	114.93**	–	− 30.39
		TN_g_	− 0.73	− 3.64	–	–	–	–	–
		TP_g_	− 7.35	–	− 0.11	–	–	–	–
Cs	2018	NRE	− 1.90	− 26.70*	–	2.17	–	-0.22***	–
		PRE	− 2.16	–	− 50.40	–	19.61**	–	− 81.05*
		TN_g_	− 0.29	− 0.98	–	–	–	–	–
		TP_g_	− 2.37	-	-0.07	–	–	–	–
	2019	NRE	− 3.55	− 33.41	–	0.58	–	-0.17*	-
		PRE	2.34	–	− 277.86**	–	19.89*	-	− 39.89*
		TN_g_	− 2.78**	3.99	–	–	–	–	–
		TP_g_	− 1.25	–	− 0.03	–	–	–	–

Sb: *Stipa breviflora*; Cs: *Cleistogenes songorica.*

SM: soil moisture; STN: soil total nitrogen; STP: soil total phosphorus; TN_g_: green leaf nitrogen; TP_g_: green leaf phosphorus; TN_s_: senesced leaf nitrogen; TP_s_: senesced leaf phosphorus.

Reported data correspond to correlation coefficients r with *p* values represents as bold asterisk (*, ** and *** indicate 0.01 < *p* < 0.05, 0.001 < *p* < 0.01, and *p* < 0.001, respectively).

**Figure 3:**
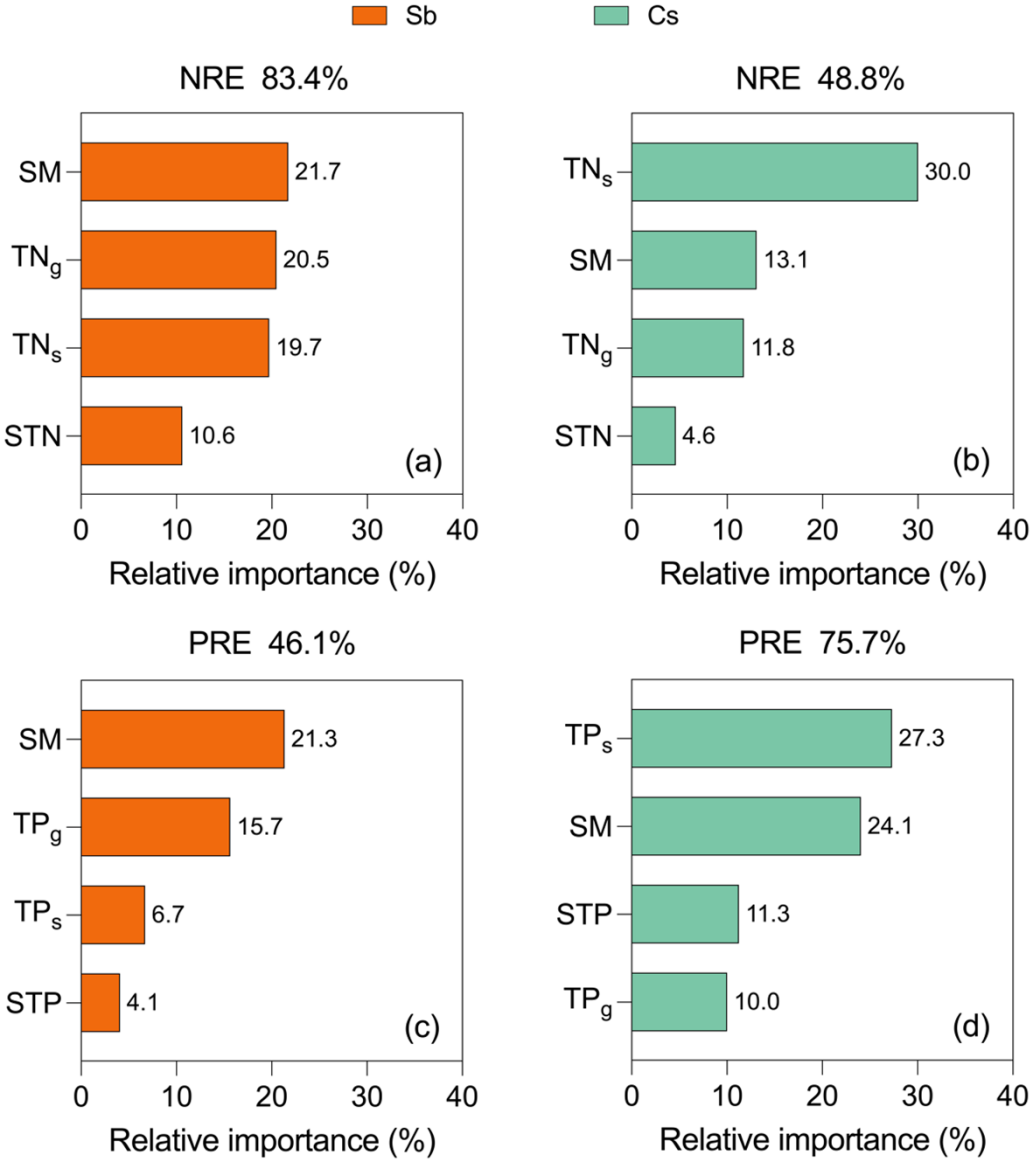
Random Forest to rank the relative importance of influence factors on NRE (**a** and **b**) and PRE (**c** and **d**) in two dominant species. Sb: *Stipa breviflora*; Cs: *Cleistogenes songorica*. SM: soil moisture; STN: soil total nitrogen; STP: soil total phosphorus; TN_g_: green leaf nitrogen; TP_g_: green leaf phosphorus; TN_s_: senesced leaf nitrogen; TP_s_: senesced leaf phosphorus.

According to the results of Random Forest analysis, soil properties and leaf nutrients explained 83.4% and 46.1% of the variation in NRE and PRE of *S. breviflora* (Fig. 3a, c) as well as 48.8% and 75.7% of the variation in NRE and PRE of *C. songorica*, respectively (Fig. 3b, d)*.* Of which the soil moisture (explained 21.3–21.7%) and senesced leaf nutrient (explained 27.3–30.0%) are the most important variables influencing nutrient resorption in *S. breviflora* and *C. songorica*, respectively.

## Discussion

The close association between the green leaf nutrition content (e.g., N and P), which is highly vulnerable to anthropogenic and environmental disturbances, and leaf functions, as well as plant nutrient utilization strategies has compelling attention in nutrient-limited terrestrial ecosystems^1,6,28^. Compared to grazing prohibition (CK), moderate grazing (MG) significantly enhanced leaf N and P content in *C. squarrosa* supporting the hypothesis that grazing would increase leaf nutrient concentration (*p* < 0.05; Fig. 1b, d). This was also corroborated for dominant species in other grassland types, e.g., typical, meadow and Tibetan alpine steppe^12,29,30^, which considered to be associated with plastic environment responses and shifts in intraspecific trait variability, particularly the soil environment. However, it is still controversial how the local soil conditions impact leaf chemical traits (e.g., N and P) in grassland ecosystems. While many studies have recognized the close relationship between soil physicochemical properties and leaf nutrients, e.g., studies in semi-arid grassland revealed that soil N and moisture were positively correlated with leaf N content^31^. Conversely, our findings revealed that foliar nutrients remained unaffected by soil variables (Table 2), consistent with the previous study conducted in the desert grassland^32^. This is due partly to foliar chemical traits that is conditional influenced by soil properties, but consistently dominated by the effects of intraspecific trait variability^30^. The higher foliar nutrients recorded in *C. squarrosa* under MG may be attributed to its overcompensation mechanism resulting in aboveground regrowth (i.e., lower C:P and N:P ratios; Supplementary Table S1)^33^ and shifts in nutrient reallocation^34,35^ to reduce biomass loss. Theoretically, leaf chemical traits in the dominant species *S. breviflora* should respond to MG in the same way but partly differ owing to its low palatability (Fig. 1)^30,36^. For instance, the green leaf P in *S. breviflora* showed no response to MG (Fig. 1c) attributed to its low risk of herbivory resulting in nutrient utilization as well as adaptive strategies that are barely responsive to grazing (i.e., stable C:P and N:P ratios; Supplementary Table S1)^33^. Moreover, leaf N in *S. breviflora* partially showed a similar response as *C. squarrosa*, but lower N after grazing with interannual differences probably due to the inhibition of protein synthesis as a result of specific environment changes or climate events (e.g., drought stress; Table 1)^37^.

It is noteworthy that the plant strategies are linked to grazing intensity, e.g., our results suggested that green leaf N in *C. squarrosa* was increased by heavy grazing (HG), while P did not exhibit the same effect (*p* < 0.05; Fig. 1 b, d). How plants’ adaption strategy balances the trade-off between tolerance (e.g., regrowth) and defensive (e.g., physical and chemical defense substances) ability responses to selection pressures of herbivory is valuable to injury further. Ma et al. revealed that *A. frigida* exhibit chemical defense responses to overgrazing^38^. Similarly, *C. squarrosa* may be pressured by HG to produce more chemical defense substances with the carbon-based compounds rich in N to inhibit massive defoliation which in turn enhanced the N content (Fig. 1b)^39^. Unexpectedly, however, nutrients in *S. breviflora* were largely stable with increasing grazing intensity (Fig. 1a, c) due to the lower risk of herbivory and greater tolerance to disturbances^38,40,41^. As suggested by our results, foliar nutrient status significant response to grazing is coupled with plants’ adaption strategies, their interactions make it complicated to predict plant nutrient storage and utilization strategies and require further study.

Nutrient resorption, an internal mechanism for nutrient conservation, can effectively reduce plants' reliance on soil nutrient supply by minimizing nutrient loss. This adaptation enables plants to thrive in barren environments such as desert steppes^1,9,42^. Numerous studies have indicated that grazing impacts nutrient resorption^8–10^, however, the co-regulation of grazing intensity or even plant traits on it remains largely unstudied. Our findings demonstrated that NRE in *S. breviflora* and *C. squarrosa* was enhanced by MG compared to CK (Fig. 2), which corresponds with the hypothesis. Nevertheless, studies in alpine meadows displayed conflicting results^13^. Foraged species will generate its overcompensation mechanism to reduce biomass loss if abundant N is provided^34,35^, which is comparatively achieved through accelerated NRE than root uptake in low nutrient bioavailable sites^9,43^. Building on the ongoing low palatability and weak responses of *S. breviflora* to selective feeding, it is sensible to assume the enhanced NRE is a result of soil nutrient depletion following the rapid growth of other foraged species^44,45^. In contrast to NRE, PRE exhibited no response to MG in both species (Fig. 2c, d), which contradicted the findings reported in the semi-arid steppe^9^ and subalpine pasture^46^ as well as the hypothesis. Disturbed leaves sometimes immobilize rather than cycle P to develop resistance^47^, additionally, relatively stable access to P may also contribute^11^. Certainly, resorption efficiency has different responses to grazing intensity, e.g., HG decreased NRE and PRE of *C. squarrosa* in this study (Fig. 2). It was also corroborated by the findings of Wang et al. in semiarid grassland^12^. This phenomenon may be attributed to the presence of more refractory N/P-containing compounds produced by plants as a defense mechanism against excessive grazing pressure^39,48^. While there have been advances toward improving our understanding of plant internal nutrient cycle response to grazing, questions remain to be addressed as the regulation process of soil conditions and foliar traits on nutrient resorption since diverse conclusions have been drawn.

Soil conditions (e.g., nutrient status and soil moisture) were detected to have a close relationship with nutrient resorption efficiency^7,49^, in some cases, even more so than genetics^50^. Soil moisture is estimated to be the relatively most important factor affecting NRE and PRE in *S. breviflora* (Fig. 3), which is observed to have a negative correlation in this paper (Table 2). A similar result has been reported by Ren et al. in semi-arid steppe^21^. In general, increased soil moisture is conducive to substrate decomposition and soil microbial activity, particularly in arid environments, thereby significantly enhancing nutrient bioavailability in soil^24,25^. Simultaneously, we found that soil N and NRE in *S. breviflora* were negatively correlated (Table 2), some studies have also corroborated that increased soil nutrients reduce resorption efficiency conditionally in natural environments^20,21^. Thus, enhanced soil moisture was supposed to release pressure on resorption efficiency in species which are responsive to changes in water, e.g., *S. breviflora* (C3 species). However, this is not a uniform conclusion. A positive correlation between soil moisture and resorption efficiency was observed in Loess Plateau^51^. Moreover, no correlation was found in a semi-arid region^26^ and Mediterranean climate zone^52^, as was the case for *C. squarrosa* in this study which is unpredicted as the hypothesis (Table 2). Inconsistencies seem to stem from divergent responses of plants, due to the differences of both physiological and/or structural, to soil conditions^26^. For instance, C4 species (e.g., *C. squarrosa*) were detected with relatively high intrinsic water use efficiency and insensitive to water deficits or even some water-related processes (Table 2)^53,54^.

Foliar chemical traits (e.g., N and P), relative to soil resources, is also a critical driver of nutrient resorption efficiency^28^. Resorption efficiency (NRE and PRE) was found to be adversely linked with green leaf nutrients in a meta-analysis^27^ and literature review^28^. However, this association has not been uniformly supported. It was reported that resorption efficiency was positively related to leaf nutrient in the semi-arid regions^55^ and Mediterranean^56^. Our study also found a similar relationship in *S. breviflora* (Table 2). This evidence points to a positive influence of foliar nutrients on resorption efficiency in barren ecosystems^56–58^ which is contrary to our hypothesis. Higher foliar nutrients were approved capable of withdrawing a higher percentage of active compounds with N and P related to enzymatic activity, photosynthesis and even metabolism (e.g., photosynthetic enzymes and pigments) from leaves^55,59–62^, which is prerequisites and may induce higher resorption efficiency. However, NRE in *C. squarrosa* was found not correlated with green leaf N (Table 2), which was consistent with the conclusions of Aerts^63^ and Güsewell^60^. Solubilized N is not restricted during the resorption process may account for the contradiction, which depends on the flow of solutes from senescing tissues and may be facilitated by abundant osmotically active substances in the senescing biomass^60,64,65^. Differing from *S. breviflora*, the senesced leaf nutrients were the predominant factor that determined resorption efficiency in *C. squarrosa* as analyzed by randomForest (Fig. 3). The incomplete nutrient resorption (e.g., senescent leaf N > 10 mg/g, P > 0.5 mg/g, Fig. 1)^7,66,67^, relative to the calculation method, is supposed to be the key driver. It was associated with the complicated local environment, such as frost damage^12,68^ and even drought^7,69^, which disrupted the process by irreversible tissue damage or even leaf death and ultimately reduced nutrient resorption efficiency^70^. However, it needs to be verified further owing to a lack of sufficient evidence.

In summary, our results partially supported the hypothesis that moderate grazing enhanced green leaf nutrient (N and P) content and NRE in *C. songorica*. *S. breviflora*’s NRE and partly green leaf N content showed a similar pattern to moderate grazing, while PRE in both species did not respond to that. Heavy grazing consistently increased N content but reduced NRE and PRE in *C. songorica*, however, P content in *C. songorica* as well as N and P content in *S. breviflora* were largely stable unexpectedly. Soil moisture negatively influenced both NRE and PRE as hypothesized which is considered to be the most dominant driver of nutrient resorption efficiency in *S. breviflora*. Moreover, for *S. breviflora*, soil N is adversely linked with NRE, and green leaf nutrients are positively associated with NRE and PRE. Senesced leaf nutrients were estimated as the predominant factor influencing nutrient resorption in *C. songorica*. Our findings suggest that although *C. songorica* has stronger drought resistance and nutrient reuse capacity in response to grazing, *S. breviflora* with a conservative resource use strategy would be more advantageous in nutrient storage in the stressed desert steppe.

## Materials and method

### Site description

**Figure 4:**
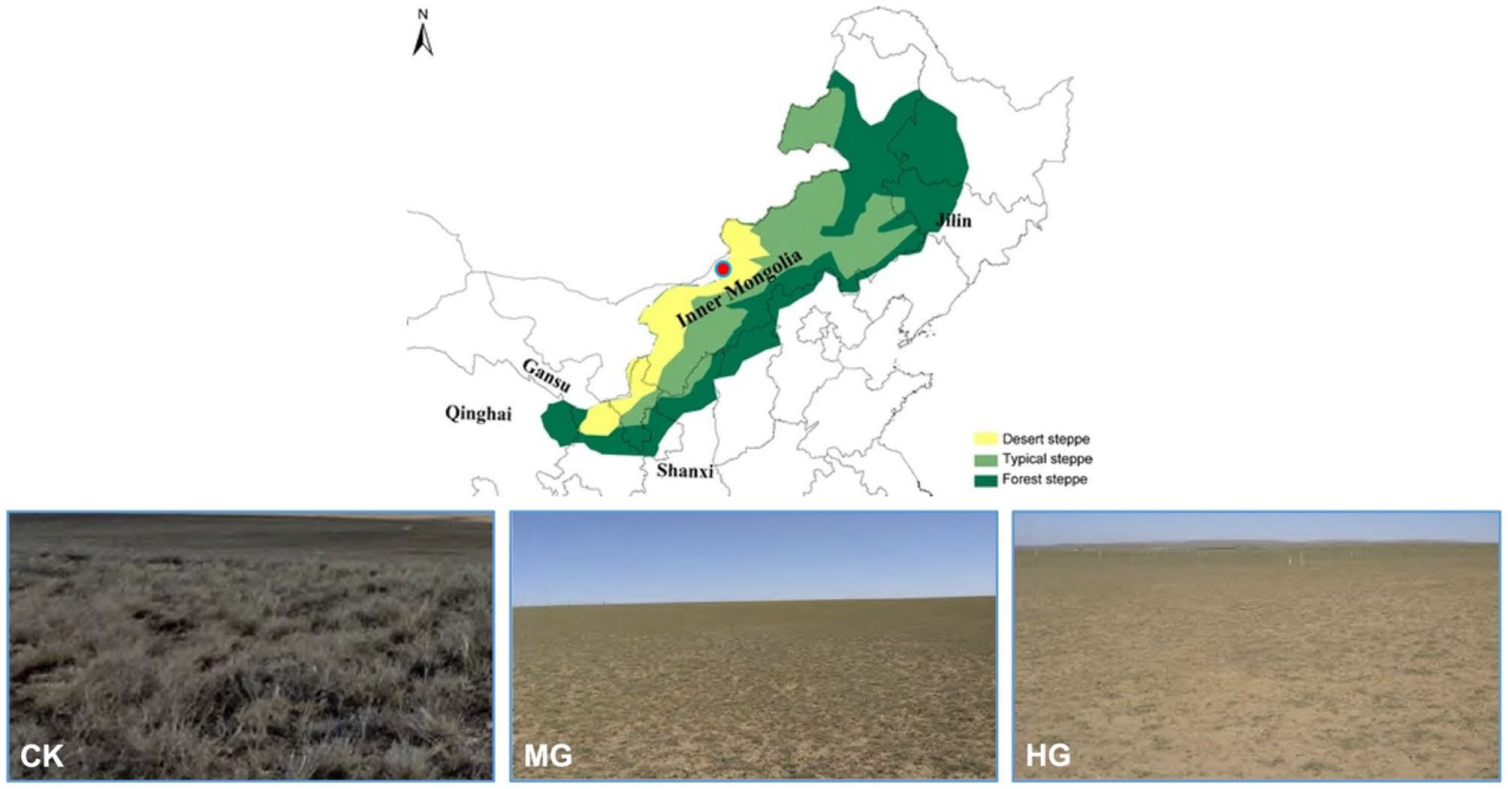
Location of the study site and photographs of grazing intensity treatments (CK: control (no grazing); MG: moderate grazing, and HG: heavy grazing).

The research was conducted in Siziwang Banner, central Inner Mongolia, northern China (41°47′17″ N, 111°53′46″ E, elevation 1450 m) (Fig. 4). The region has a typical semiarid climate^71^, which has been classified as a severe drought area^72^ with an average annual precipitation of 220 mm (from 2002 to 2019) which mainly concentrated from May to September. The average annual temperature is 3.7 °C, with a 175-day frost-free season. Soil in this region is dominated as Kastanozem (as classified by the FAO) with sandy loam in texture^73^. The vegetation coverage is no more than 20%, with less than 25 species of plant diversity. *Stipa breviflora* (C3 grass) and *Cleistogenes songorica* (C4 grass) are the dominant perennial grasses in the experimental site.

### Experimental design

The region was grazed intensely in history^74^. The experiment site of about 40 ha was established in June 2002 and designed to include three grazing intensities with six randomly located 2.2 ha replicate plots per treatment (Fig. 4). The three grazing intensities were based on local carrying capacities^75^: control check (CK) (fenced to exclude animal grazing), moderate grazing (MG) 1.82 sheep (ha half yr)^−1^ (corresponding to 4 sheep per plot), and heavy grazing (HG) 2.71 sheep (ha half yr)^−1^ (corresponding to 6 sheep per plot). The plots were grazed from June to November with 2-year-old wethers weighing around 30 kg, which were replaced every three years. Each day, the animals were brought to the plots for free grazing starting at 6 am and returned back to shelter for water and salt supplementation at 6 pm.

### Sampling and properties measurements

*Cleistogenes songorica* (*C. squarrosa*) and *Stipa breviflora* (*S. breviflora*) were identified and collected according to their morphology and specimens deposited in Herbarium of the Institute of Botany, Chinese Academy of Sciences (can be searched with Chinese Virtual Herbarium official website https://www.cvh.ac.cn/). For each plot, at least 30 shoots of each species with similar growth and showing no signs of herbivory or disease were randomly selected in August, the fully unfolded upper third and fourth leaves in vegetative branches were sampled, then mixed into a composite green leaf sample. From September to October, the remaining leaves on each branch were monitored weekly, the completely dried and yellowed but still attached leaves were sampled and then mixed into a composite senesced leaf sample of each species per plot. Before chemical analysis, composite samples of each species were oven-dried for 48 h at 65 °C and then finely ground with a ball mill. Total leaf carbon and nitrogen concentration were determined by CN analyzer (Vario TOC Elemental Inc, Germany). Total leaf P concentration was measured by concentrated sulfuric acid/perchloric acid oxidation and followed by ammonium molybdate colorimetric analysis^76^.

Within each of the experimental plots, three soil cores (5 cm diameter) were randomly collected from a maximum depth of 10 cm and mixed into a composite sample in August. Composite samples were air-dried and sieved to 2 mm to remove roots and tiny rocks before chemical analysis. Total soil N content was determined on a CN analyzer (Vario TOC Elemental Inc, Germany). Soil total P was measured using the alkali-molybdenum antimony colorimetric method^77^. Soil moisture (0–10 cm) was measured by mass loss of soil on heating (105 °C).

### Data analyses

Nutrient resorption efficiency (RE), defined as the proportion of the senesced leaf nutrient pool that is resorbed, was calculated as the nutrient (N and P) concentrations in the green and senesced leaves for corresponding species and plots, with the following formula^78^:

$${\text{Re}} \left( \% \right) = \left[ {1 - \left( {{\text{TN}}_{{\text{s}}} /{\text{TN}}_{{\text{g}}} } \right)} \right] \times 100\%$$

where TN_g_ and TN_s_ present the nutrient (i.e., N and P) concentrations in green and senesced leaves, respectively.

All data analyses were performed in R studio (Team RC 2019, R Core Team 2019; Vienna, Austria). One-way ANOVAs (n = 6) were used to analyze differences linked to grazing intensity treatments in: leaf nutrient content; nutrient resorption efficiency; ratio of leaf nutrients (C:P and N:P); and soil properties (soil moisture, soil total N and P). Means were then compared using Tukey’s multiple comparison test (*p* < 0.05). Relationships among soil properties, foliar nutrient content and nutrient resorption efficiency as well as leaf nutrient concentrations were observed by linear regression. In addition, Random Forest (RF) is a non-parametric method that is composed of numerous tree models trained from bootstrap samples of the data^79^. RF can rank the predictor variable's relative importance according to the regression prediction error of out-of-bag^80^. We used the randomForest package^81^ to evaluate and rank the variables that influence nutrient resorption efficiency in each species.

### Ethical approval

The authors declare that they have no known competing financial interests or personal relationships that could have appeared to influence the work reported in this paper.

### Rights and permissions

*Stipa breviflora* and* Cleistogenes songorica* are not endangered wild species, and widely distributed in the experimental area.The collection of studied plant samples does not need permission, and we have the absolute right to manage the experimental region and take samples.

### Statement

The experiment complied with relevant institutional, national, and international guidelines and legislation. Cuiping Gao and Guodong Han from the Key Laboratory of Grassland Resources, Ministry of Education, College of Grassland, Resources and Environment, Inner Mongolia Agricultural University undertook the formal identification of the plant material used in this study. Samples were deposited in the Key Laboratory of Grassland Resources, Ministry of Education, College of Grassland, Resources and Environment, Inner Mongolia Agricultural University, not in a publicly available herbarium.

### Supplementary Information

Supplementary Table S1.

## Data Availability

The datasets generated and/or analyzed during the current study were available from the corresponding author upon reasonable request.
