# Supplementary material for: Different responses of foliar nutrient resorption efficiency in two dominant species to grazing in the desert steppe
Source: Sci Rep. 2024 Feb 19;14:4090. doi: 10.1038/s41598-024-53574-3 (PMC10876624; doi:10.1038/s41598-024-53574-3)
Supplement: Supplementary file 1 — Supplementary Table S1. [file 41598_2024_53574_MOESM1_ESM.pdf]

Supplementary Table S1. Ratio of green leaf nutrients in two species as a function of grazing intensity. Data correspond to mean values ± standard errors (n=6). Different bold lowercase letters represent significant differences among grazing intensities (p<0.05).

Year	Grazing	Sb		Cs	
		C:P	N:P	C:P	N:P
2018	CK	469.3±66.8**b**	23.5±2.6**b**	321.1±19.3**ab**	17.5±1.8**b**
	MG	458.8±43.0**b**	25.4±1.5**b**	275.6±25.4**b**	17.2±1.6**b**
	HG	653.9±28.4**a**	32.8±1.7**a**	361.7±56.4**a**	22.5±4.0**a**
2019	CK	479.7±32.2	20.2±2.0	368.0±12.4**a**	15.6±0.9**b**
	MG	483.6±10.6	18.1±0.6	269.0±13.6**b**	14.6±0.5**b**
	HG	488.2±81.4	18.4±2.8	362.0±35.2**a**	16.9±1.5**a**

CK: control (no grazing); MG: moderate grazing (1.82 sheep ha^{-1} corresponding to 4 sheep per plot); HG: heavy grazing (2.71 sheep ha^{-1} corresponding to 6 sheep per plot).

Sb: *Stipa breviflora*; Cs: *Cleistogenes songorica*.
